# Community engagement to manage acute malnutrition: implementation research in Kupang district, Indonesia

**DOI:** 10.2471/BLT.18.223339

**Published:** 2019-07-04

**Authors:** Blandina Rosalina Bait, Jee Hyun Rah, Airin Roshita, Roberth Amaheka, Vama Chrisnadarmani, Maria Reneldys Lino

**Affiliations:** a United Nations Children’s Fund, Kupang Field Office, Jalan Polisi Militer 2, Kupang, East Nusa Tenggare Province, Indonesia.; b United Nations Children’s Fund, Jakarta, Indonesia.; c Kupang District Health Office, Kupang, Indonesia.

## Abstract

**Objective:**

To improve the low coverage and performance of a programme on community-based management of acute malnutrition, implemented between October 2015 and April 2018 in Kupang district in rural Indonesia.

**Methods:**

To investigate why the coverage and performance were low in the first year of the programme, we conducted a semiquantitative evaluation between August and September 2016. We used the results from the evaluation to inform programme improvement, by developing and modifying community mobilization strategies. We employed a multipronged approach to improve community awareness on acute malnutrition and on community-based services for such condition. This approach involved workshops, focus discussion groups in the community and sensitization events at health posts that had issues with community engagement. Community health workers increased their efforts in active case finding by visiting households with children who had missed the community health post sessions. We measured the performance using three Sphere minimum standard performance indicators: proportion of children recovering (> 75%); defaulting (< 15%); and dying (<10%).

**Results:**

The community mobilization efforts increased the screening rate from 17% (564/3278) in October 2015 to 66% (6793/10 251) in March 2018. In 2017, the programme met the three performance indicators: 79% (256/326) of children recovered; 10% (34/326) defaulted; and less than 1% (2/326) died.

**Conclusion:**

In Indonesia, community mobilization is central for addressing severe acute malnutrition in children younger than five years. This strategy includes securing political leadership and effective messaging alongside locally tailored strategies and continuous ground-level support.

## Résumé

**Objectif:**

Améliorer la couverture et les performances d'un programme de gestion communautaire de la malnutrition aiguë, mis en œuvre entre octobre 2015 et avril 2018 dans le district rural de Kupang, en Indonésie.

**Méthodes:**

Afin de déterminer les raisons pour lesquelles la couverture et les performances du programme étaient faibles durant sa première année, nous avons réalisé une évaluation semi-quantitative entre août et septembre 2016. Nous avons utilisé les résultats de cette évaluation pour favoriser l'amélioration du programme, en élaborant et en modifiant des stratégies de mobilisation de la communauté. Nous avons adopté une démarche pluridimensionnelle pour sensibiliser davantage la communauté à la malnutrition aiguë et aux services communautaires permettant d'y remédier, avec notamment des ateliers, des groupes de discussion dans les communautés et des activités de sensibilisation dans les postes sanitaires qui avaient eu des problèmes au niveau de l'engagement de la communauté. Les agents de santé communautaires ont redoublé d'efforts pour dépister les cas, en se rendant dans les foyers composés d'enfants qui avaient manqué les séances organisées aux postes sanitaires. Nous avons mesuré les performances à l'aide de trois indicateurs des standards minimums Sphère: proportion d'enfants qui se sont rétablis (> 75%); qui n'ont pas suivi le programme (< 15%); qui sont décédés (< 10%).

**Résultats:**

Les efforts de mobilisation de la communauté ont permis d'augmenter le taux de dépistage, passé de 17% (564/3278) en octobre 2015 à 66% (6793/10 251) en mars 2018. En 2017, le programme a atteint les trois indicateurs de performance: 79% (256/326) des enfants se sont rétablis; 10% (34/326) n'ont pas suivi le programme; et moins de 1% (2/326) sont décédés.

**Conclusion:**

En Indonésie, la mobilisation de la communauté est cruciale pour lutter contre la malnutrition aiguë sévère chez les enfants de moins de cinq ans. Cette stratégie exige d'avoir une direction politique ainsi qu'une communication efficace, en même temps que des stratégies adaptées au contexte local et le soutien continu de la population.****

## Resumen

**Objetivo:**

Mejorar la deficiente cobertura y el rendimiento de un programa de gestión comunitaria de la malnutrición aguda, implementado entre octubre de 2015 y abril de 2018 en el distrito de Kupang, en la zona rural de Indonesia.

**Métodos:**

Para investigar por qué la cobertura y el rendimiento fueron deficientes en el primer año del programa, realizamos una evaluación semicuantitativa entre agosto y septiembre de 2016. Utilizamos los resultados de la evaluación para informar sobre la mejora del programa, mediante el desarrollo y la modificación de las estrategias de movilización comunitaria. Empleamos un enfoque múltiple para mejorar la concienciación de la comunidad sobre la malnutrición aguda y sobre los servicios basados en la comunidad para este tipo de afecciones. Este enfoque incluyó talleres, grupos focales de discusión en la comunidad y eventos de sensibilización en los puestos sanitarios que tenían problemas con la participación de la comunidad. Los trabajadores comunitarios de salud incrementaron sus esfuerzos en la búsqueda activa de casos visitando hogares con niños que no habían asistido a las sesiones del puesto sanitario de la comunidad. Medimos el rendimiento utilizando tres indicadores de rendimiento estándar mínimos de Esfera: proporción de niños que se recuperan (> 75 %), que no asisten (< 15 %) y que mueren (< 10 %).

**Resultados:**

Los esfuerzos de movilización comunitaria aumentaron la tasa de evaluación del 17 % (564/3278) en octubre de 2015 al 66 % (6793/10 251) en marzo de 2018. En 2017, el programa cumplió los tres indicadores de rendimiento: 79 % (256/326) de los niños recuperados; 10 % (34/326) no asistieron; y menos del 1 % (2/326) murieron.

**Conclusión:**

En Indonesia, la movilización de la comunidad es fundamental para abordar la malnutrición aguda grave de los niños y niñas menores de cinco años. Esta estrategia incluye garantizar el liderazgo político y la eficacia de los mensajes, junto con estrategias adaptadas a las necesidades locales y el apoyo continuo en la zona.

## ملخص

**الغرض:**

تحسين التغطية المحدودة والأداء المنخفض برنامج حول الإدارة المجتمعية لسوء التغذية الحاد، والذي تم تنفيذه بين أكتوبر/تشرين أول 2015 وأبريل/نيسان 2018 في منطقة كوبانغ بريف إندونيسيا.

**الطريقة:**

لاستكشاف سبب محدودية التغطية وانخفاض مستوى الأداء في السنة الأولى من البرنامج، أجرينا تقييماً شبه كمي بين أغسطس/آب وسبتمبر/أيلول 2016. واستخدمنا نتائج التقييم لتحسين البرنامج عن دراية، وذلك بواسطة تطوير استراتيجيات التحفيز المجتمعي وتعديلها. وقمنا بتوظيف أسلوب متعدد الجوانب لتحسين الوعي المجتمعي بسوء التغذية الحاد والخدمات المجتمعية لمثل هذه الحالة. شمل هذا الأسلوب ورش العمل، ومجموعات المناقشة المركزة في المجتمع، وأحداث التوعية في الوظائف الصحية التي كانت لها مشاكل مع المشاركة المجتمعية. قام العاملون في مجال الصحة المجتمعية بزيادة جهودهم في إيجاد الحالات النشطة عن طريق زيارة الأسر التي بها أطفال لم يحضروا الجلسات اللاحقة للصحة المجتمعية. قمنا بقياس الأداء باستخدام ثلاثة مؤشرات قياسية للأداء الأدنى: نسبة الأطفال الذين يتعافون (> 75٪)؛ والمتدهورين (< 15٪)؛ والمحتضرين (< 10٪).

**النتائج:**

أدت جهود التحفيز المجتمعي لزيادة معدل الفحص من 17٪ (564/3278) في أكتوبر/تشرين أول 2015 إلى 66٪ (6793/10251) في مارس/آذار 2018. في عام 2017، حقق البرنامج مؤشرات الأداء الثلاثة: 79٪ (256/326) من الأطفال تعافوا؛ و10٪ (34/326) منهم تدهورت حالته؛ وتعرض أقل من 1٪ (2/326) للوفاة.

**الاستنتاج:**

يعد التحفيز المجتمعي في إندونيسيا أمراً جوهرياً لمواجهة سوء التغذية الحاد المتأخر لدى الأطفال الأقل من خمس سنوات. تتضمن هذه الاستراتيجية تأمين القيادة السياسية والرسائل الفعالة إلى جانب الاستراتيجيات الموضوعة محلياً والدعم المتواصل على الأرض. ****

## 摘要

**目的:**

旨在改善 2015 年 10 月至 2018 年 4 月在印度尼西亚古邦农村地区实施的、以社区为基础的急性营养不良管理项目覆盖率低和绩效不佳的情况。

**方法:**

为了调查该项目首年覆盖率低、绩效不佳的原因，我们在 2016 年 8 月至 9 月期间进行了半定量评估。我们制定并调整了社区动员策略，使用此次评估中得出的结果进行项目改善。我们采用多管齐下的方法来提高急性营养不良的社区意识以及针对此情况的社区服务水平。这一方法包括在面临社区参与难题的社区内部举办研讨会、焦点小组访谈和在卫生站开展相关宣传活动。社区医务工作者通过走访那些错过社区卫生站会议的、有孩童的家庭，加大积极发现病例的力度。我们使用三个领域的最低标准绩效指标来进行衡量：康复儿童比例（大于 75%）、未接受治疗儿童比例（小于 15%）和死亡儿童比例（小于 10%）。

**结果:**

社区动员工作令筛检率从 2015 年 10 月的 17%（564/3278）提高到 2018 年 3 月的 66%（6793/10251）。2017 年，该项目达到了三项绩效指标要求：79%（256/326）的儿童康复；10%（34/326）的儿童未接受治疗；只有不到 1%（2/326）的儿童死亡。

**结论:**

在印度尼西亚，社区动员是解决 5 岁以下儿童严重急性营养不良问题的关键。该战略包括政治领导、有效的信息传达、因地制宜的策略与持续的基层支持。****

## Резюме

**Цель:**

Повышение охвата и эффективности программы по привлечению общественности к решению проблемы острого недоедания, которая проводилась в период с октября 2015 года по апрель 2018 года в районе Купанг, Индонезия.

**Методы:**

Для того чтобы понять причины низкого охвата населения и эффективности программы в первый год ее применения, авторы провели приближенно-количественную оценку в период с августа по сентябрь 2016 года. Результаты оценки использовались для совершенствования программы за счет разработки и изменения стратегий мобилизации общественности. Использовался комплексный подход к улучшению представления в обществе об остром недоедании и общественных возможностях помощи в таких случаях. Данный подход предусматривает проведение семинаров, обсуждений в рамках целевых групп внутри сообщества и разъяснительных мероприятий в пунктах оказания медицинской помощи там, где не удавалось в должной мере привлечь общественность. Общественные медицинские работники активизировали усилия по выявлению фактических случаев недоедания, посещая семьи с детьми, не явившиеся на обязательные мероприятия в местных пунктах оказания медицинской помощи. Авторы измеряли показатели эффективности программы по трем минимальным стандартным индикаторам в данной области: по доле детей, которые выздоровели (более 75%), не показали улучшения (менее 15%) или умерли (менее 10%).

**Результаты:**

Усилия по мобилизации общественности привели к увеличению охвата скринингом с 17% (564 из 3278 человек) в октябре 2015 года до 66% (6793 из 10 251 человека) в марте 2018 года. В 2017 году программа соответствовала всем трем показателям эффективности: 79% (256 из 326) детей выздоровели, 10% (34 из 326) не показали улучшения и менее 1% (2 из 326) умерли.

**Вывод:**

Привлечение общественности в Индонезии играет основную роль в разрешении проблемы острого тяжелого недоедания среди детей младше пяти лет. Стратегия включает обеспечение вовлечения политических лидеров и эффективное информирование населения в рамках специализированных стратегий, учитывающих местные условия, при постоянной поддержке на местах

## Introduction

Despite decades of significant economic growth, Indonesia has the fourth highest burden of acute malnutrition in the world.^1^ Of the country’s 23 million children who are younger than five years, nearly 805 000 are affected by severe acute malnutrition.^1^^,^^2^ Children with severe acute malnutrition are 11 times more likely to die than those who are well nourished^3^^–^^5^ and this type of malnutrition is the most dangerous form of child malnutrition.^3^^–^^5^

While treatment of severe acute malnutrition has been a standard component of health services for many years in Indonesia,^6^ the health ministry reported that only 20 000 children with severe acute malnutrition received treatment in 2017 and that the quality of facility-based care was low.^7^ The World Health Organization recommends that children aged 6–59 months with severe acute malnutrition and no medical complications can be given outpatient treatment, known as community-based management of acute malnutrition.^4^^,^^8^ This approach has maximized the coverage and successful treatment of children with severe acute malnutrition in other countries.^9^^,^^10^ Therefore, the Indonesian health ministry, together with United Nations Children’s Fund (UNICEF) and Action Against Hunger, piloted an implementation model for community-based management of acute malnutrition in rural eastern Indonesia between October 2015 and April 2018.^8^ The aim of the pilot was primarily to demonstrate and build an evidence base on how to integrate community-based services for management of acute malnutrition into routine health service delivery in Indonesia.

At the outset, UNICEF in collaboration with the health ministry, Kupang District Health Office and Action Against Hunger developed and implemented a simplified community-based management of acute malnutrition protocol and training package. Key programme components included community mobilization; case detection and referral through active case finding; medical screening at health-care facilities to decide on inpatient or outpatient treatment for severe acute malnutrition; and outpatient care for severe acute malnutrition without medical complications using ready-to-use therapeutic food.^4^^,^^8^ For the initial severe acute malnutrition screening, community health workers measured children’s mid-upper arm circumference and all children whose mid-upper arm circumference was below 12.5cm, visibly thin or had bilateral oedema were referred to local health-care facilities for confirmation of severe acute malnutrition. Subsequently, facility-based health workers confirmed the diagnosis by a weight-for-height z-score < −3 SD and/or mid-upper arm circumference < 11.5cm and/or presence of bilateral oedema.^4^^,^^8^ UNICEF and Action Against Hunger have made efforts to strengthen the capacity of the health ministry, provincial and district health office authorities, and the women’s empowerment organization on the community-based management of acute malnutrition protocol.^8^ The women’s empowerment organization consisted of the wives of local government officials, and had been proactively advocating for addressing various health and nutrition issues including acute malnutrition.

In the first year of implementation, however, the programme had low coverage, high default rates and slow response of malnourished children to treatment. Monitoring data collected between October 2015 and December 2016 highlighted several key challenges in programme coverage and quality. Specifically, the proportion of children screened was estimated to be low, since only 14% (640/4597) of eligible children younger than five years of age were registered in the community health post, known as *Posyandu*. Furthermore, only a quarter of children were being effectively followed up after being identified with severe acute malnutrition. The average proportion of children recovering was low (44%) and the average proportion of children defaulting (that is, not returning for follow-up visits for two or more consecutive weeks) was high (48%).^1^

Here we describe the efforts made to improve programme coverage and quality, focusing on a semiquantitative evaluation of access and coverage to investigate underlying issues, and subsequent actions taken to develop and modify community mobilization strategies.

## Methods

### Setting

The community-based management of acute malnutrition programme was intensified in six subdistricts of Kupang district in East Nusa Tenggara province, the southernmost of Indonesia’s 34 provinces. We selected these subdistricts in consultation with the local government considering the number of children with severe wasting and presence of nutrition staff in the health-care facilities. Of the 2.2 million children living in the province, many live in rural settings.^11^ In the province, child malnutrition is widespread and wasting affects 15% (92 537/616 917) of children.^1^ In Kupang district, 35% (16 016/45 760) of children younger than five years of age are wasted due to multiple factors, including sub-optimal feeding practices of infants and young children, and poor water, sanitation and hygiene conditions.^1^ In 2015, a studied showed that only 13% (52/406) of children aged 6–23 months received a minimum acceptable diet, that is, children who received foods from four or more food groups during the previous day. Nearly half the households were either moderately or severely food insecure and more than a quarter of households were still obtaining water from open surface sources or unprotected well (UNICEF and Action Against Hunger, unpublished data, February 2015).

Existing primary health centres at the village and subdistrict levels provide outpatient services for acute malnutrition and health workers in these centres are trained on community-based management of acute malnutrition. Community health workers (CHWs) provide health services at health posts once a month. These posts are used as platform for monthly growth monitoring for children younger than five years of age, including screening for severe acute malnutrition. Between these monthly events, CHWs visit each household to follow up with families and provide additional services as needed.

### Semiquantitative evaluation

We conducted a semiquantitative evaluation of access and coverage in two selected subdistricts between August and September 2016 to identify key factors contributing to the low coverage and performance of the programme. We evaluated whether a supportive supervision mechanism for health workers and CHWs or whether a nearby outpatient therapeutic centre (less than 2 km) contributed to programme coverage above 50%.

A team of field enumerators deployed by Action Against Hunger collected the data, using a combination of quantitative and qualitative approaches. The quantitative approach focused on reviewing and analysing monthly governmental routine data on the community-based management of acute malnutrition, submitted by the six subdistricts, and interviewing caregivers of defaulters. The qualitative approach focused on assessing the community awareness on malnutrition, capacity of the health workers and CHWs, parental caring practices and health service quality. We conducted 21 focus groups discussions and 44 semi-structured and informal interviews to collect qualitative data from the CHWs, health workers, caregivers and key community stakeholders. To maximize the geographical representativeness, participants for the focus group discussions and in-depth interviews were selected based on their residential location and distance from the outpatient therapeutic centres. The evaluators identified participants from the registration data available at the community health outpost and through discussions with the local village leaders and key stakeholders. To collect necessary information in a standardized manner, staff members from Action Against Hunger and UNICEF, in consultation with the government, developed and pre-tested the structured discussion and interview guides before the focus group discussions and in-depth interviews. Each participant provided a written informed consent before the discussion or the interview. To help participants feeling safe and comfortable, the discussion took place in a secure private venue and efforts were made to build a rapport by engaging in informal conversations before the focus group discussions and in-depth interviews. We employed a simplified lot quality assurance sampling to test whether an association existed between supportive supervision or distance and programme coverage.

Across the two subdistricts, the evaluation showed that the average coverage for the outpatient therapeutic programme was low (25%; 95% confidence interval:17–33). The combined results from the evaluation methods indicated that the primary cause of defaulting was the distance from the outpatient therapeutic site. After seeing some improvements in their severely malnourished child’s condition, caregivers stopped bringing their children to the outpatient therapeutic centre to avoid the long distance of travel, waiting at the outpatient therapeutic site and travel costs. Most caregivers considered 2 km as far, while the distance of the closest and the farthest villages to the outpatient therapeutic sites ranged from 0.1–10 km.

Importantly, the focus discussions and the interviews revealed that knowledge and awareness on the risks of severe acute malnutrition, admission and discharge criteria, and inpatient and outpatient treatment protocol was relatively poor among health workers and CHWs. Furthermore, health workers and CHWs were lacking confidence in performing their daily tasks and responsibilities for the community-based management of acute malnutrition programme, including severe acute malnutrition screening and confirmation, provision of the outpatient treatment and counselling the caregivers. Overall, the study results suggested that a supportive supervision mechanism was effective to address the low performance of health workers and CHWs, however such mechanism did not seem to improve access and coverage of the programme.

The results also highlighted the lack of community awareness on acute malnutrition and community-based services for management of acute malnutrition as the key contributing factor to the low monthly attendance rate for severe acute malnutrition screening at a community health post and poor engagement in the community-based programme (UNICEF and Action Against Hunger, unpublished data, September 2016). Specifically, the group discussions and in-depth interviews showed that most people were aware of the high prevalence of malnutrition in their community, however, they rarely recognized severe acute malnutrition as a serious condition requiring medical treatment. Furthermore, the caregivers were usually the mothers, but the fathers were the primary decision-maker of the households and their knowledge and awareness on malnutrition was poor.

### Implementation of strategies

The results of the semiquantitative evaluation informed the priorities for 2017 and beyond for the programme. The priorities were to intensify efforts to improve programme coverage and performance by strengthening community engagement and active case findings done by the CHWs, along with capacity building of health facility workers and CHWs. We analysed the semiquantitative evaluation data by each subdistrict to develop strategies for areas which had a high proportion of defaulters (at least 50%) and less than 50% of children recovering, and children attending the monthly community health post. In addition, UNICEF and Action Against Hunger conducted quarterly in-depth programme review meetings to track progress against the semiquantitative evaluation results.

To improve community engagement for services on severe acute malnutrition and community-based management of acute malnutrition, the district health office and UNICEF adopted a multipronged approach, including workshops, community sensitization events and communication messages.^12^^,^^13^ The approach was first implemented in 12 community health posts across six subdistricts, with low attendance rate and was later extended to other health posts. To map key community stakeholders on health and nutrition issues, the government, UNICEF and Action Against Hunger asked mothers, caregivers, families and key community stakeholders about the most reliable and respected source of information on health and nutrition issues. Between January 2017 and February 2018, government authorities and the women’s empowerment organization held workshops on community mobilization for parents, government authorities, religious leaders and community leaders. In each subdistrict and selected villages, field staff from Action Against Hunger conducted focus group discussions for two to three hours with village authorities, such as chief of villages, religious leaders, teachers, health staff and CHWs. The aim of these discussions was to improve the authorities’ awareness and enhance their commitment towards addressing severe acute malnutrition and the programme.^12^^,^^13^ We prepared a wide range of communication materials including leaflets, videos and posters, with key messages on signs of severe acute malnutrition, importance of screening children for severe acute malnutrition, and bringing children to the monthly community health post. We also prepared messages on the benefit of the community-based services for management of acute malnutrition and on encouraging parents, families and communities to help identify children with severe acute malnutrition. 

CHWs visited households with children younger than five years of age and invited mother and fathers to attend community sensitization events on malnutrition and its effect, and the importance of attending monthly community health post. Community leaders also attended the sessions. The events were held in two community health posts in each subdistrict and we prioritized community health posts with low attendance rate, and a high proportion of children younger than 5 years of age. Parents and caregivers of children that had recovered from severe acute malnutrition were encouraged to share positive experiences during these events. Information about community-based services for management of acute malnutrition were announced through multiple platforms, such as church services and village coordination meetings. Local religious leaders and members of the women’s empowerment organization actively participated in community mobilization efforts by attending each community mobilization session and joining the severe acute malnutrition screening efforts made at the community health outpost. Health workers and CHWs made home visits in their catchment area every month to remind parents and caregivers to attend the monthly community health post for participation in severe acute malnutrition screening. We organized various recreational events, such as dancing, movie and theatre performance, at the community health post to improve community interest and participation in the monthly health outreach event.

The CHWs increased their efforts in active case finding by visiting households with children who had missed the community health post sessions (according to the official record of the community health post) and screening these children for severe acute malnutrition. Specifically, we targeted communities with an average community health post attendance rate below 60% per month for active case finding. Based on the initial screening at home, using mid-upper arum circumference, CHWs closely followed children with possible severe acute malnutrition and referred them to nearby health facilities for confirmation of severe acute malnutrition. The CHWs repeatedly visited households with children with possible severe acute malnutrition to encourage the parents and family to bring the child to a nearby health clinic for confirmation of severe acute malnutrition. Occasionally, the CHWs had to facilitate the arrangement of transportation to enable the caregivers to bring the malnourished child to a nearby health clinic.

The district government and the women’s empowerment organization introduced radio talk shows to generate public awareness on severe acute malnutrition, community-based services for management of acute malnutrition, ready-to-use therapeutic food and other relevant issues.

Progress and challenges of the implementation of the community-based management of acute malnutrition were monitored through joint visits with government and other partners to the intervention area. Concurrent efforts were made by the health ministry and the district health office to integrate community-based management of acute malnutrition into the existing health services, which included capacity development through trainings of district government, health staff and CHWs on programme monitoring.

To assess outcomes, we used three out of the four Sphere Project performance indicators, that is, > 75% of children recover, default < 15% children defaulting, and < 10% of children dying.^14^

## Results

**Fig. 1:**
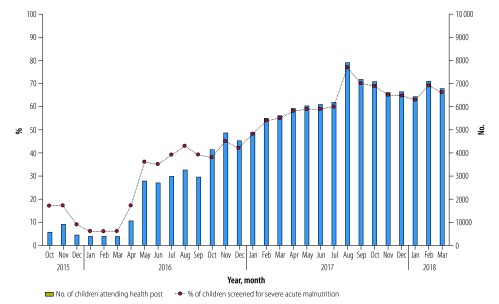
Attendance at community health post and screening for severe acute malnutrition of children younger than five years, Kupang district, Indonesia, October 2015 to March 2018

Intensive community mobilization efforts resulted in improved monthly community health post attendance, which led to an increased screening rate from 17% (564/3278) in October 2015 to 66% (6793/10 251) in March 2018 (Fig. 1). The greatest improvement in attendance rate was seen in 12 community health posts that had issues with community engagement, the average rate increased from < 50% in October 2015 to 79% in March 2018. In addition, the intensified efforts to follow up children with potential severe acute malnutrition after the initial screening improved the proportion of children with confirmed severe acute malnutrition, from no children turning up for confirmation in October 2015 to 70% (112/160) in March 2018.

Between October 2015 and March 2018, more than 6500 children were screened on average for severe acute malnutrition each month and 719 children were admitted for treatment. For the year 2017, the programme consistently met three out of four sphere minimum standard performance indicators: the average proportion of children recovering was 79% (256/326); the average proportion of children defaulting was 10% (34/326); and deaths were less than 1% (2/326; Table 1).

**Table 1:** Performance indicators for a programme on community-based management of acute malnutrition in Kupang district, Indonesia, 2015–2018

Outcome	Sphere minimum standard, %^a^	Year and month																
		2015			2016						2017						2018	
		Oct–Dec, no. (%) *n* = 9	Average %		Jan–Mar, no. (%) *n* = 58	Apr–Jun, no. (%) *n* = 82	Jul–Sep, no. (%) *n* = 40	Oct–Dec, no. (%) *n* = 70	Average %		Jan–Mar, no. (%) *n* = 74	Apr–Jun, no. (%) *n* = 88	Jul–Sep, no. (%) *n* = 95	Oct–Dec, no. (%) *n* = 69	Average %		Jan–Mar, no. (%) *n* = 75	Average %
Children recovered	> 75	1 (11)	11		25 (43)	31 (38)	17 (43)	45 (64)	47		59 (80)	76 (88)	68 (72)	53 (77)	79		50 (67)	67
Children defaulting^b^	< 15	8 (89)	88		23 (40)	43 (52)	21 (53)	23 (33)	45		8 (11)	5 (6)	13 (14)	8 (12)	11		9 (12)	12
Children who died	< 10	0 (0)	0		0 (0)	0 (0)	0 (0)	0 (0)	0		1 (1)	0 (0)	1 (1)	0 (0)	< 1		0 (0)	0

^a^ Sphere minimum standard is the percentage for which the outcome of the programme is deemed successful.

^b^ The numbers include both unconfirmed and confirmed defaulters. A child was classified as a defaulter if the child had missed two consecutive visits for 2 weeks.

Owing to UNICEF’s strong advocacy, the province and district governments and the women’s empowerment organization fully committed to supporting continued implementation of the programme. This was achieved by leveraging their own resources and scaling up this essential life-saving intervention to the remaining 18 subdistricts in Kupang district and as well as to an additional 21 districts/municipalities in East Nusa Tenggara Timur province.

Moreover, due to the successful implementation of the programme in Kupang district, three other districts in East Nusa Tenggara province adopted the programme and have been implementing it since 2018. Furthermore, 10 districts in the same province plan to introduce community-based services for management of acute malnutrition, by integrating these into the existing health system in 2019.

## Discussion

The experiences and the findings described here highlight the critical role of community mobilization in enhancing the performance of programmes addressing severe acute malnutrition. The intensification of the community mobilization strategy, which ranged from village to subnational level, secured political commitment from decision-makers, and leveraged additional infrastructure, human and financial resources to support the programme. This effort led to enhanced ownership and support for the programme by the community. A thorough understanding of the context where community-based management of acute malnutrition is being implemented is essential for developing locally relevant and tailored approaches.^15^ While the strategy adopted to intensify community mobilization achieved positive results, these efforts require continuous re-enforcement to sustain the results.

Our programme experience is comparable to that of other countries. A study in Pakistan and Ethiopia also identified barriers related to access to treatment for severe acute malnutrition, including the distance to health facility, high economic costs for caregiving, knowledge of services, knowledge of malnutrition and child’s refusal of ready-to-use foods.^16^ Another study conducted in rural Bihar, India, emphasized the importance of engaging communities for a successful community-based management of acute malnutrition programme, by including local disease concepts and beliefs in health promotion strategies, and by improving community awareness that undernutrition is a treatable disease.^15^

The community-based management of acute malnutrition programme implemented in Kupang District has had positive effects in addressing severe acute malnutrition in a limited high-risk area. Efforts are underway by the health ministry and UNICEF to integrate this strategy into revised national policy and programme guidelines; and translate lessons learnt to local approaches that can reach children with severe acute malnutrition across Indonesia.

This implementation model has proved to be sustainable, with the provincial and district governments in East Nusa Tenggara having allocated funding to support capacity building activities, to procure ready-to-use therapeutic food and anthropometry devices, and to scale up the services to additional districts. For 22 districts, the province health office has developed a roadmap for programme scale up and has established a task force with budgetary funding to support programme implementation. In April 2019, the health ministry decided to scale up the integrated management of acute malnutrition services across 260 of the country’s 514 districts by 2020, making this evidence-based life-saving intervention more accessible and available to the most vulnerable groups of children. A costing analysis will be conducted in 2019 to estimate the total cost of treatment per child and to understand the cost–effectiveness of the community-based management of acute malnutrition in the Indonesian context.

## Conclusion

Community mobilization is central for addressing severe acute malnutrition in children younger than five years of age, and thus, a continual effort of community mobilization conducted at all levels is needed. Implementers interested in a community-based programme should bear in mind that no single approach fits all settings and they would need to consider locally tailored strategies for community engagement in community-based management of acute malnutrition. Securing the strong leadership of key stakeholders, such as heads of districts, women’s empowerment organizations, community and religious leaders and CHWs, is essential for strengthening community engagement, as these stakeholders are listened to and respected. Future replication of the community-based management of acute malnutrition in other regions of Indonesia, supported by a strong monitoring and evaluation mechanism, is warranted, as the current findings originate from only six subdistricts and may not be fully generalizable.
